# Research trends of facial nerve injury after cerebellopontine angle tumor: CiteSpace-based bibliometric analysis

**DOI:** 10.3389/fneur.2025.1525669

**Published:** 2025-05-07

**Authors:** Xinxin Li, Bing Bai, Beibei Nie

**Affiliations:** Department of Neurosurgery, The First Affiliated Hospital of Zhengzhou University, Zhengzhou, China

**Keywords:** cerebellopontine angle, tumor, facial nerve, CiteSpace, bibliometric analysis, research trend

## Abstract

**Background:**

A bibliometric analysis was conducted to understand the current research status and trends in facial nerve injury after cerebellopontine angle (CPA) tumors to identify new perspectives for future research.

**Methods:**

CiteSpace was used to visualize and analyze relevant literature included in the CNKI and WanFang databases, and the Web of Science Core Collection from 2015 to 2024. Chinese literature was deduplicated using NoteExpress.

**Results:**

A total of 7,021 studies was retrieved, showing a pattern of rapid increase in this research area over the past 10 years. Protection and management of the facial nerve in surgery and early recognition of facial nerve injury were the research hotspots and trends.

**Conclusion:**

This study emphasizes the importance of intraoperative protection and management of the facial nerve. Limited research has addressed the postoperative facial nerve injury from the perspective of functional rehabilitation and patient psychology. These areas need more attention and focused research.

## 1 Introduction

The cerebellopontine angle (CPA) is a triangular three-dimensional area located in the anterolateral aspect of the posterior cranial fossa between the cerebellum, pons, medulla oblongata and the petrous part of the temporal bone. Approximately 10–15% of the intracranial tumors originate in this area (1). Most CPA tumors are benign and patients may present with neurological symptoms such as hearing impairment, vestibular dysfunction, and headaches (2). Treatment options include conservative treatment, radiation therapy, and surgical removal, which should be chosen based on patient preferences and clinical indicators and conditions such as physical status and tumor size (3). About 80% ~ 94% of CPA tumors are vestibular schwannomas, which reinforces surgical resection as the optimal treatment. This procedure carries a 10% ~ 60% chance of damaging the facial nerve (3–5).

Facial nerve injury, and even facial paralysis, can occur in patients because of intraoperative damage to the cranial nerves, especially the facial nerve, secondary to CPA space constraints (6, 7). Expression defects and chewing weakness may occur when patients suffer facial nerve injury (8), potentially triggering adverse emotional states such as anxiety and depression, thereby affecting their social participation (9–12). Patients with impaired or not fully rehabilitated facial nerve function will experience asymmetric facial movements (13). These sequelae significantly compromise their physical and mental health. Kenton et al. (14) conducted qualitative interviews with patients with facial nerve injuries after vestibular schwannoma to understand their distress. They found that the root of the distress was long-term facial paralysis, alteration of self-perception, and social anxiety. Therefore, it is of great significance and necessity to visualize the current status, hotspots, and frontiers of research on patients with postoperative facial nerve injury after CPA tumors.

Bibliometrics is employed to analyze literature in a specific field, aiming to explain the structure of research trends through statistical methods, lexical analysis, and citation analysis, etc. (15). In recent years, CiteSpace has been widely used for bibliometric visualization and analysis, including citations, collaborations, countries, keywords, clusters, and burst terms (16). Currently, the characteristics and management of facial nerve injuries associated with CPA tumors remain inadequately explored. Therefore, this study aimed to employ CiteSpace software and bibliometric methods to conduct a visual analysis of relevant research, to identify the research hotspots and emerging frontiers concerning facial nerve injury following surgical treatment of CPA tumors.

## 2 Methods

### 2.1 Literature sources and search strategies

We searched the CNKI and WanFang databases, as well as the Web of Science core collection. Chinese language Database was searched using the terms “lesions of the pontine cerebellar horn region,” “lesions of the pontine cerebellar horn region,” “vestibular nerve sheath tumors,” “acoustic neuromas,” “facial nerves,” “facial nerve injuries,” “facial nerve dysfunction,” “facial paralysis,” “facial nerve palsy,” and more. We also performed searches in English using the terms “cerebellopontine angle,” “neuroma,” “acoustic,” “neurofibromatosis,” “acoustic neuroma,” “vestibular schwannoma,” “facial nerve,” “facial paralysis,” “facial nerve function,” and “facial nerve palsy.” Literature was searched for the last 10 years, from 2015 to 2024. Literatures related to facial nerve injury from cerebellopontine angle tumors was included. While, literature that is grey, duplicate, or published in languages other than English or Chinese was excluded. It’s worth noting that Chinese literature refers to publications in Chinese, not necessarily by Chinese authors. Additionally, Chinese literature was imported into NoteExpress for deduplication and then into CiteSpace software for analysis.

### 2.2 Data analysis

**Figure 1:**
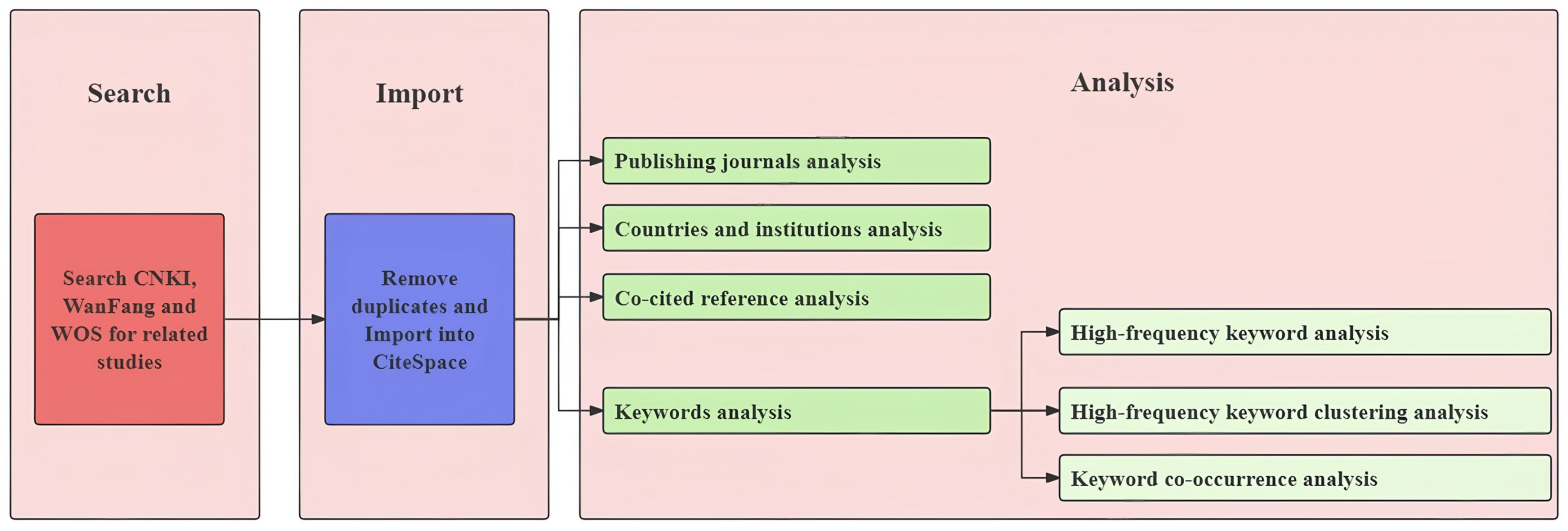
Flow chart for CiteSpace.

Chinese and English records literature were exported in RefWorks and Plain-text format, respectively, and both were imported into CiteSpace software for format conversion. The time selection used was 2015–2024, and the time slices were set to one-year intervals. Visualization and analysis were performed and plotted after the relevant parameters were set according to the software (Figure 1).

## 3 Results

### 3.1 Literature search results, publication trends, and country distribution

**Figure 2:**
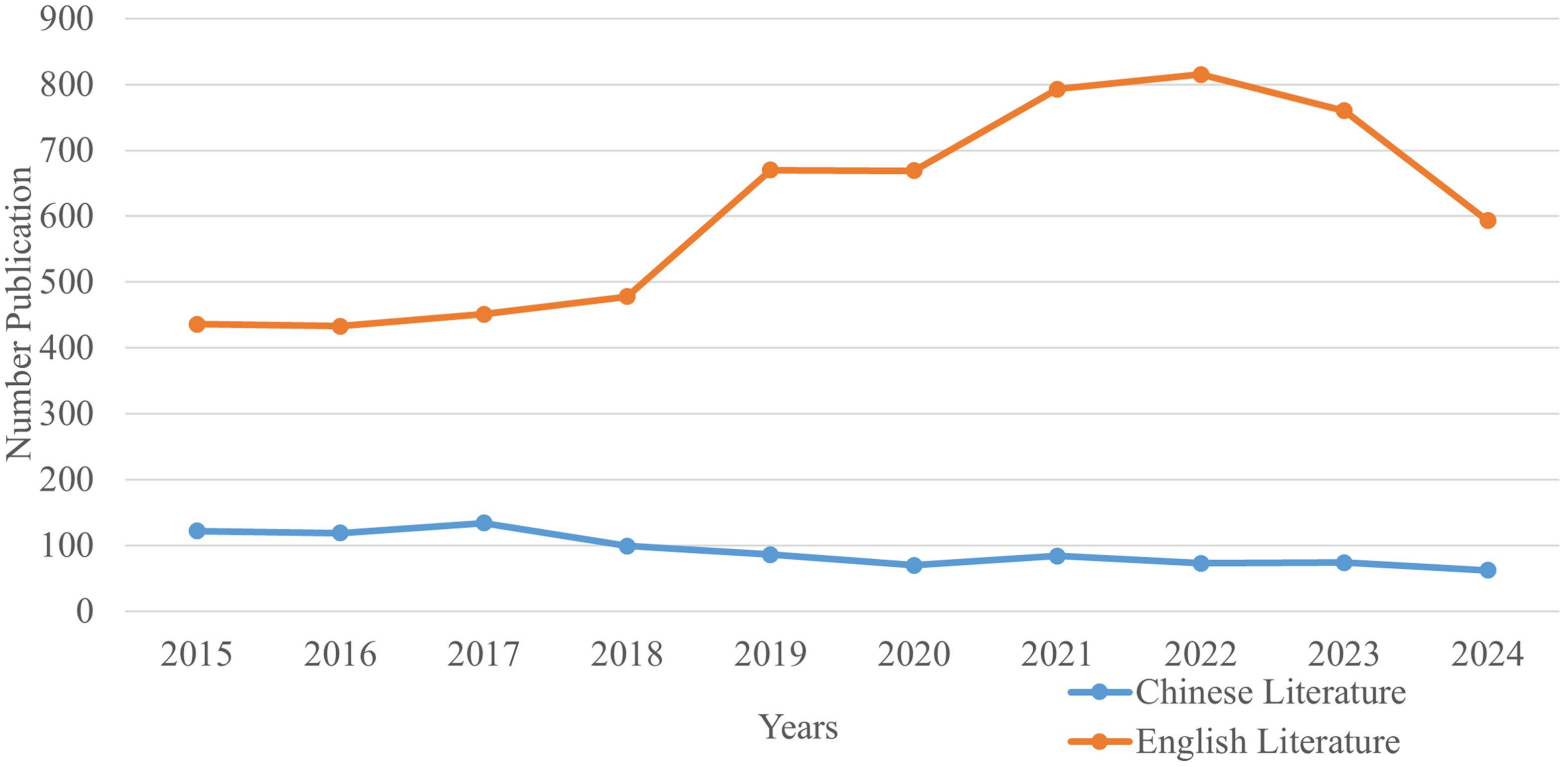
Number publication.

Altogether, 1,227 and 6,098 studies were retrieved in Chinese and English, respectively. A total of 7,021 studies remained after deduplication using NoteExpress. The number of studies related to CPA tumors and facial nerve demonstrated a decreasing trend in Chinese and an increasing trend in English (Figure 2). Furthermore, the number of Chinese studies was significantly lower than that of English. The year 2022 was the peak year for English publications on research related to the CPA and facial nerve, with 815 studies. The top 5 countries for this research, in order of publication volume, were the United States, China, Germany, Japan, and Italy. Among them, the United States ranked first with 1,703 studies, reflecting its research leadership on this field within the academic space.

### 3.2 Publishing journals analysis

Analysis was restricted to English-language journals owing to technical constraints of the bibliometric software. In terms of the number of citations, the journal *Laryngoscope* was the most cited English journal with a total of 2,349 citations for studies related to the CPA and facial nerve (Table 1).

**Table 1:** Top 5 journals in English citations.

No.	Journals	Impact factor	Citations
1	Laryngoscope	Q3/2.2	2349
2	Otolaryng Head Neck	Q1/2.6	2033
3	Otol Neurotol	Q3/1.9	1661
4	J Neurosurg	Q1/3.5	1631
5	Neurosurgery	Q1/3.9	1441

### 3.3 Countries and institutions analysis

**Figure 3:**
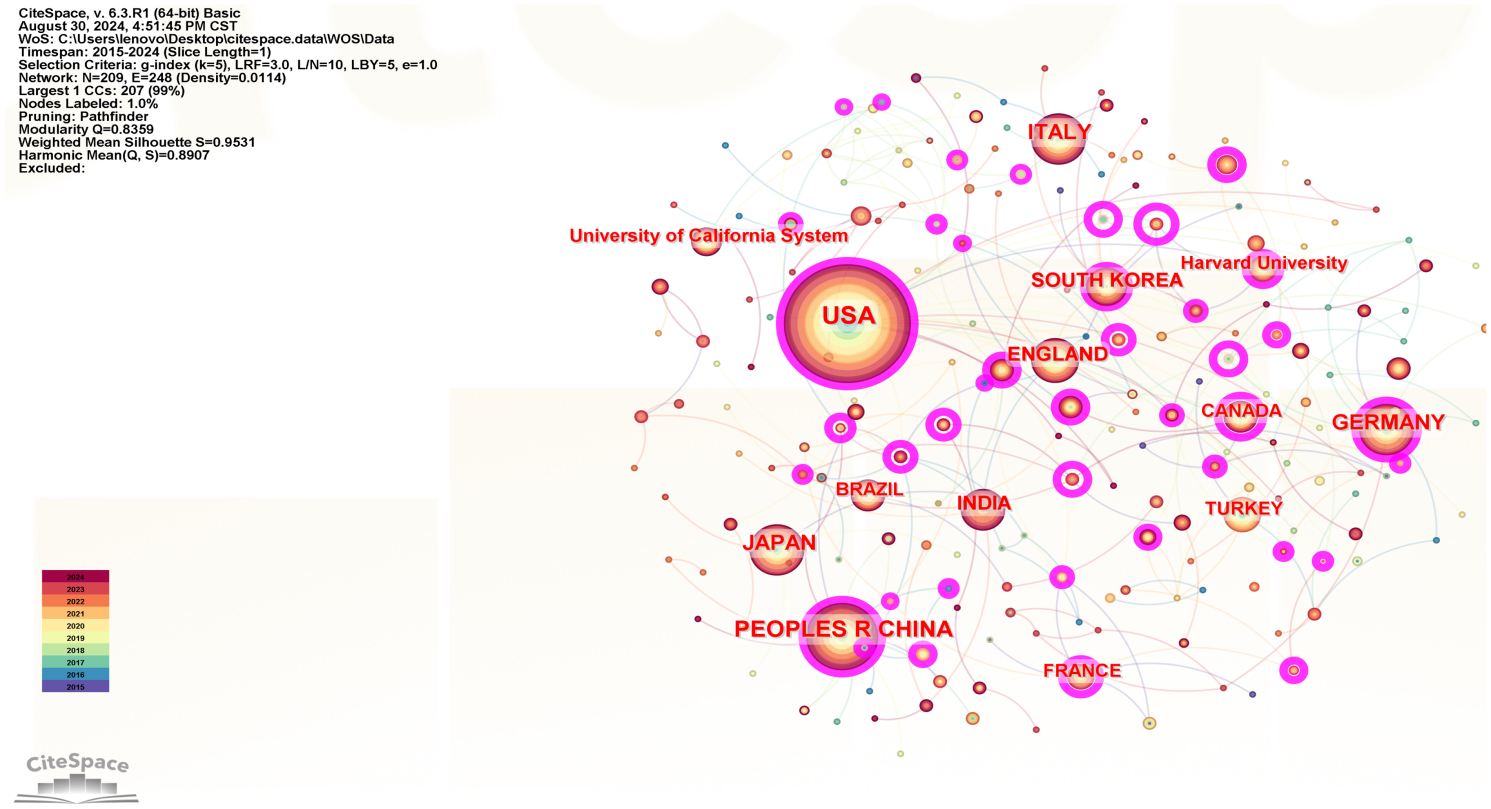
Country and institutional co-occurrence maps.

The top 5 countries were the USA, the People’s Republic of China, Germany, Japan, and Italy, while the top 5 institutions were Harvard University, the University of California System, Harvard Medical School, the University of London, and Shanghai Jiao Tong University. For details, see Figure 3 and Table 2.

**Table 2:** Top 5 countries and institutions.

No.	Count	Centrality	Co-cited reference	Count	Centrality	Institutions
1	1703	0.29	USA	161	0.37	Harvard University
2	728	0.24	Peplos Republic of China	160	0.08	University of California System
3	442	0.31	German	121	0.13	Harvard Medical School
4	438	0.06	Japan	95	0.65	University System of Ohio
5	416	0.09	Italy	87	0	Shanghai Jiao Tong University

### 3.4 Co-cited reference analysis

**Figure 4:**
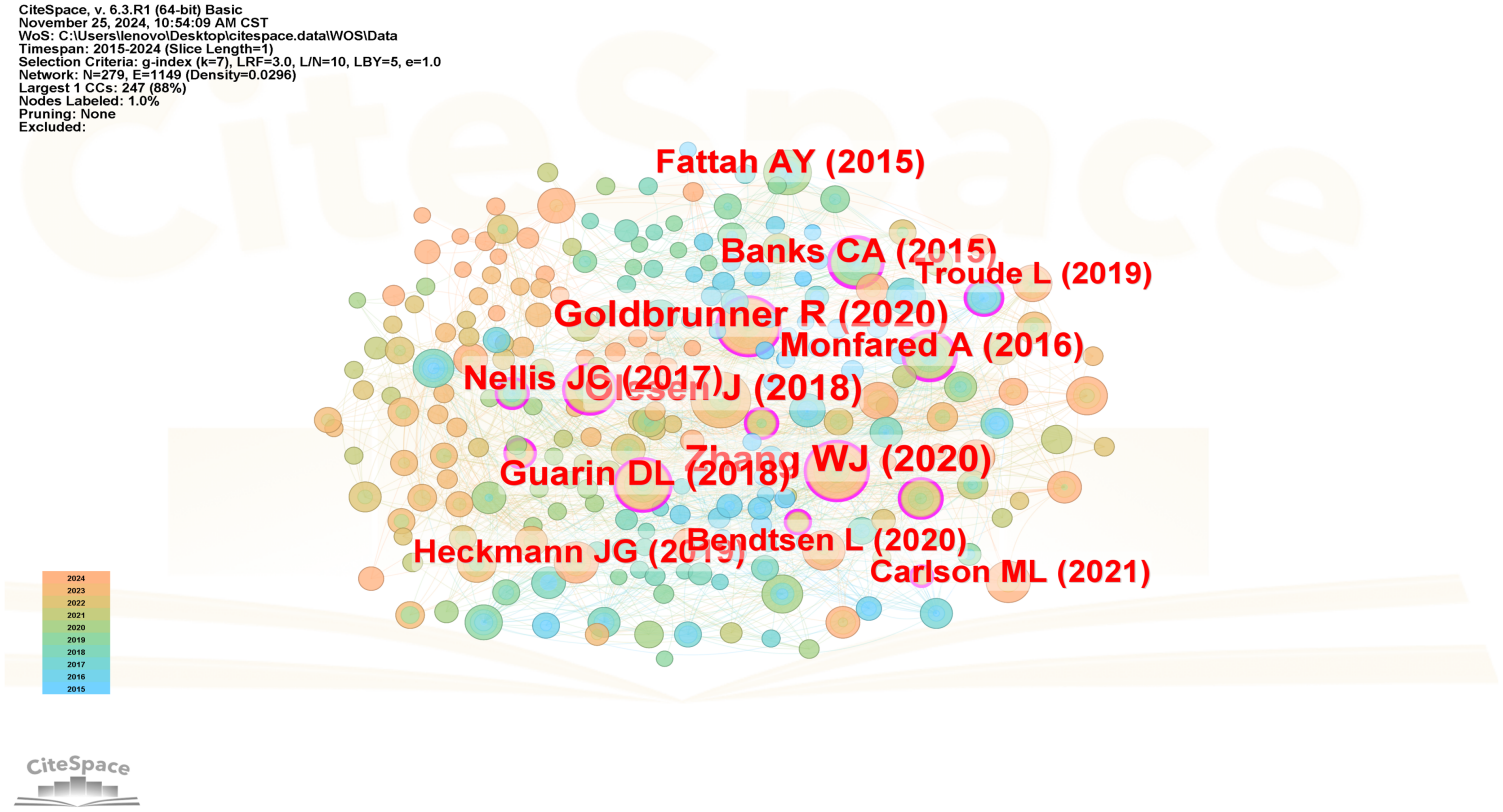
Co-cited reference map.

Table 3 presents the top ten co-cited references related to postoperative facial nerve injury in CPA tumors, which have been co-cited more than 400 times. The first co-cited reference was published in 2020 by Goldbrunner et al. (17). This article provided guidelines for the diagnosis and treatment of vestibular schwannoma and laid the foundation for conducting clinically relevant research. The second reviewed the anatomy of the facial nerve (18), and the possible etiology of facial paralysis development. The third article (19) focused on comparing the efficacy different between topiramate and acetazolamide in reducing intracranial pressure. The results showed that Topiramate was more effective. The Figure 4 shows the literature co-citation analysis.

**Table 3:** Top 10 co-cited reference.

No.	Count	Centrality	Years	Co-cited Reference
1	57	0.17	2020	Goldbrunner R, 2020, Neuro-Oncology, V22, P31, DOI: 10.1093/neuonc/noz153
2	55	0.1	2020	Zhang WJ, 2020, J Neurol, V267, P1896, DOI: 10.1007/s00415-019-09282-4
3	53	0.06	2019	W. J. Scotton, 2018, Cephalalgia, V39, P209, DOI: 10.1177/0333102418776455
4	42	0.1	2018	Guarin DL, 2018, JAMA Facial Plast Su, V20, P335, DOI: 10.1001/jamafacial.2018.0030
5	39	0.21	2015	Banks CA, 2015, Plast Reconstr Surg, V136, P223E, DOI: 10.1097/PRS.0000000000001447
6	38	0.11	2017	Nellis JC, 2017, Jama Facial Plast Su, V19, P190, DOI: 10.1001/jamafacial.2016.1462
7	38	0.21	2016	Monfared A, 2016, Neurosurgery, V79, P194, DOI: 10.1227/NEU.0000000000001162
8	36	0.07	2015	Fattah AY, 2015, Plast Reconstr Surg, V135, P569, DOI: 10.1097/PRS.0000000000000905
9	31	0.05	2019	Heckmann JG, 2019, Dtsch Arztebl Int, V116, P692, DOI: 10.3238/arztebl.2019.0692
10	28	0	2020	Bendtsen L, 2020, Lancet Neurol, V19, P784, DOI: 10.1016/S1474-4422(20)30233-7

### 3.5 Keywords analysis

#### 3.5.1 High-frequency keyword analysis

**Figure 5:**
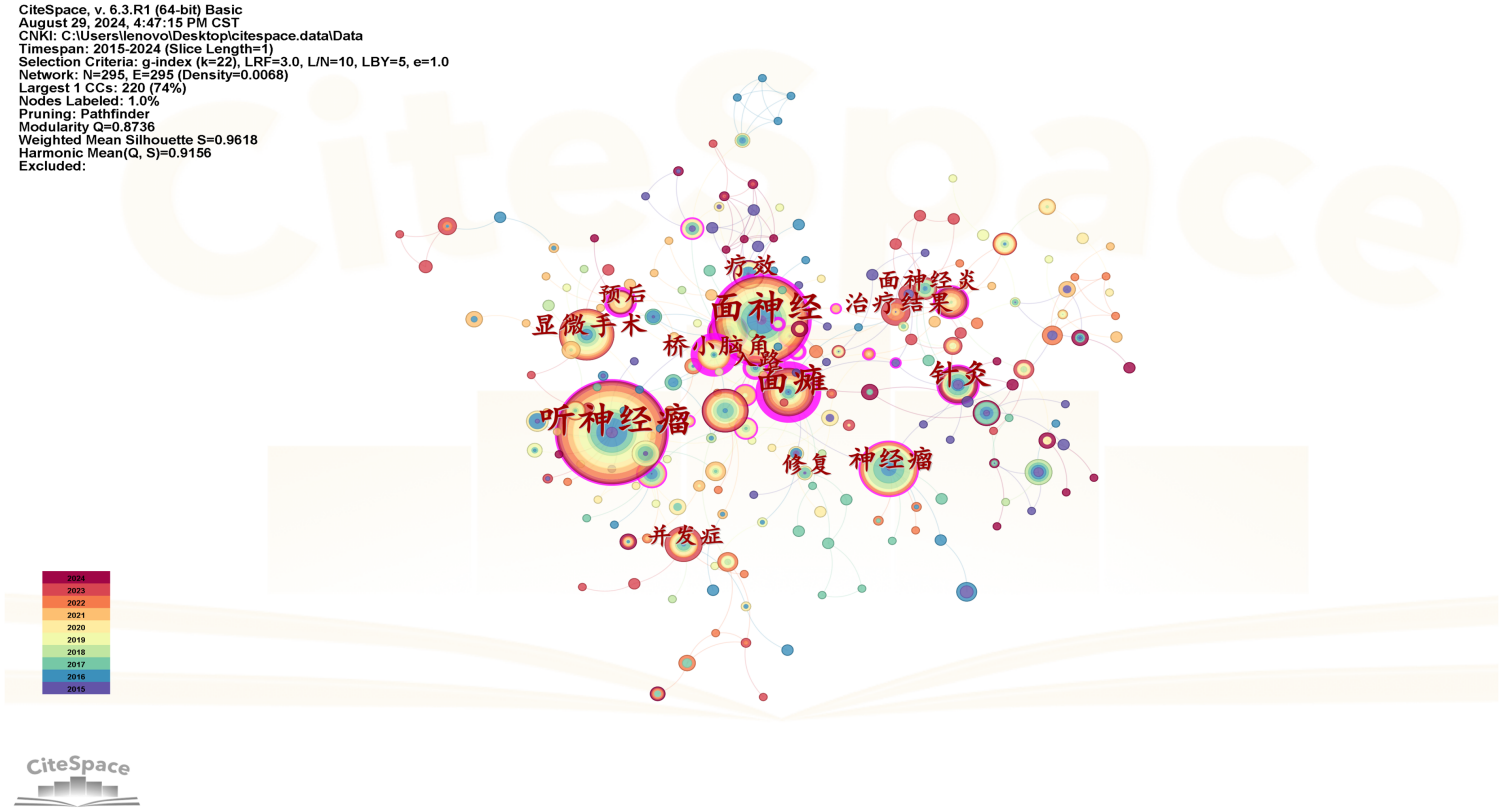
High-frequency keyword co-occurrence map of Chinese literature.

**Figure 6:**
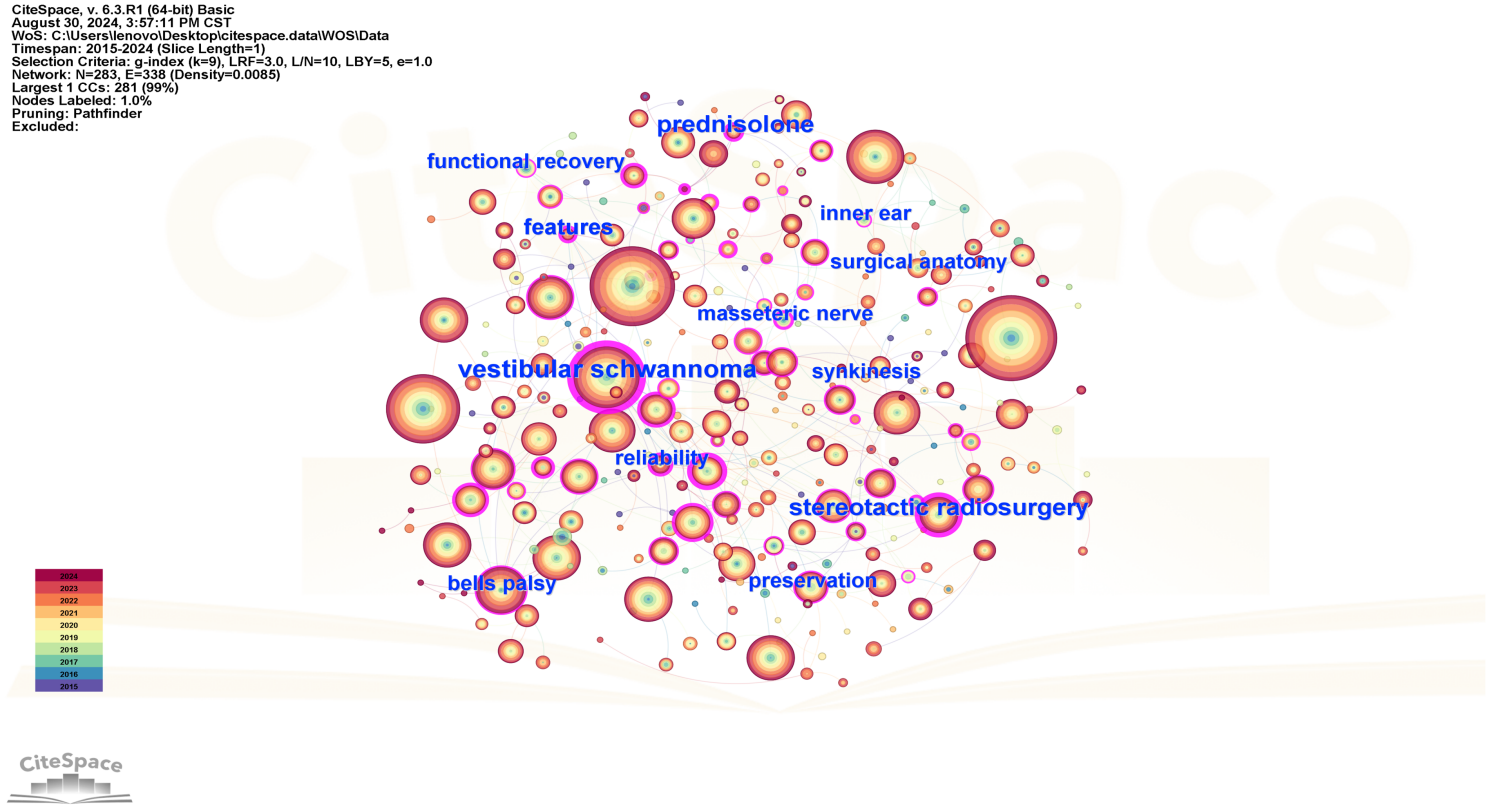
High-frequency keyword co-occurrence map of English literature.

The co-occurrence graph of high-frequency keywords in Chinese and English is shown in Figures 5, 6, after setting the time slice to 1, node type to keyword, and frequency threshold to the Top 50. Both English and Chinese studies focused on acoustic neuroma, facial nerve, facial palsy, and surgery. Table 4 shows the top ten keywords in both English and Chinese.

**Table 4:** Top 10 high-frequency keywords in English and Chinese literature.

No.	Chinese literature				English literature			
	Frequency	Centrality	Years	Keywords	Frequency	Centrality	Years	Keywords
1	191	0.17	2015	acoustic neuroma	992	0.01	2015	facial nerve
2	139	0.38	2015	Facial nerve	830	0	2015	management
3	52	0.12	2015	neuroma	637	0	2015	surgery
4	51	0.09	2015	microsurgery	561	0.72	2015	vestibular schwannoma
5	47	0.65	2015	Facial paralysis	427	0	2015	facial paralysis
6	40	0.03	2015	facial muscle spasm	317	0.11	2015	bells palsy
7	23	0.13	2015	acupuncture	307	0	2015	outcm
8	22	0.1	2015	Clinical efficacy	306	0.03	2015	paralysis
9	22	0.06	2016	complications	295	0.03	2015	acoustic neuroma
10	19	0.12	2016	neuroendoscopy	289	0.08	2015	facial palsy

#### 3.5.2 High-frequency keyword clustering analysis

**Figure 7:**
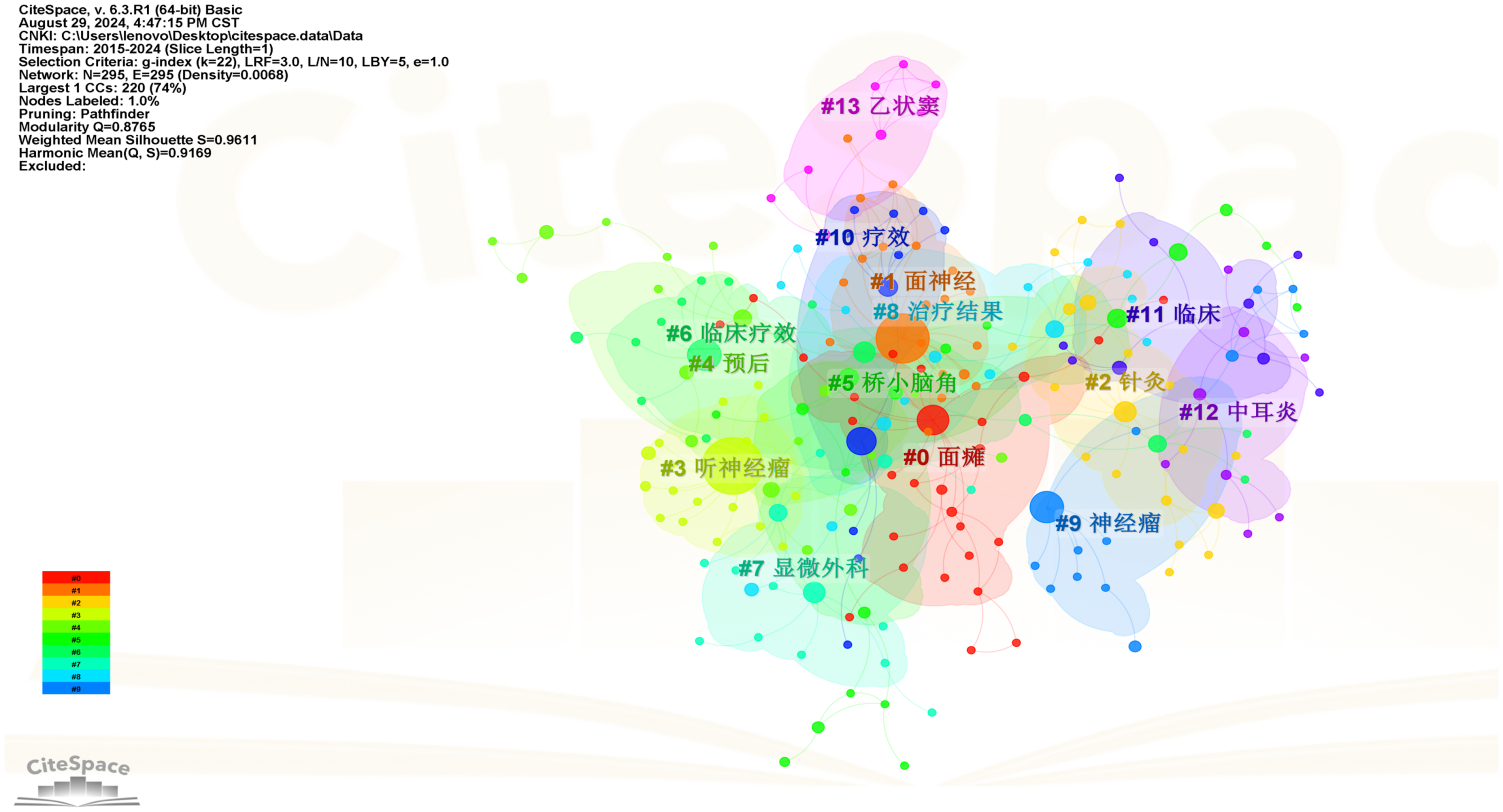
Clustering mapping of high-frequency keywords in Chinese literature.

**Figure 8:**
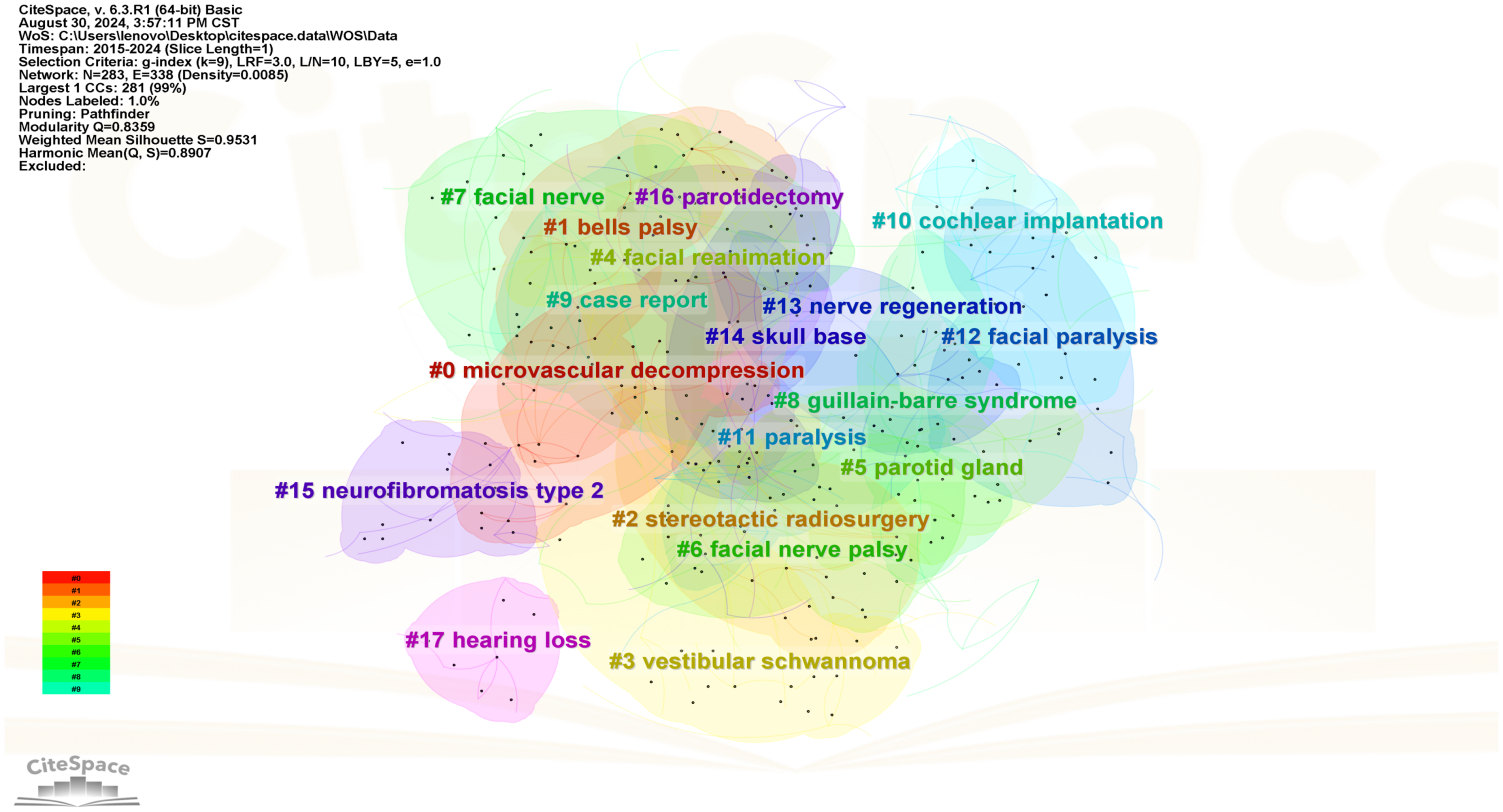
Clustering mapping of high-frequency keywords in English literature.

It is generally accepted that a Q value > 0.3 indicates a significant clustering result, while an S value > 0.5 is considered to represent reasonable clustering in keyword analysis. The Q and S values of the Chinese and English studies were 0.877 and 0.961, and 0.836 and 0.953, respectively, indicating that the cluster analysis was efficient and convincing. The smaller the cluster tag value, the more keywords it contains. The cluster analysis mapping of high-frequency keywords related to facial nerve injury and CPA tumors in Chinese and English studies is shown in Figures 7, 8, respectively.

#### 3.5.3 Keyword co-occurrence analysis

**Figure 9:**
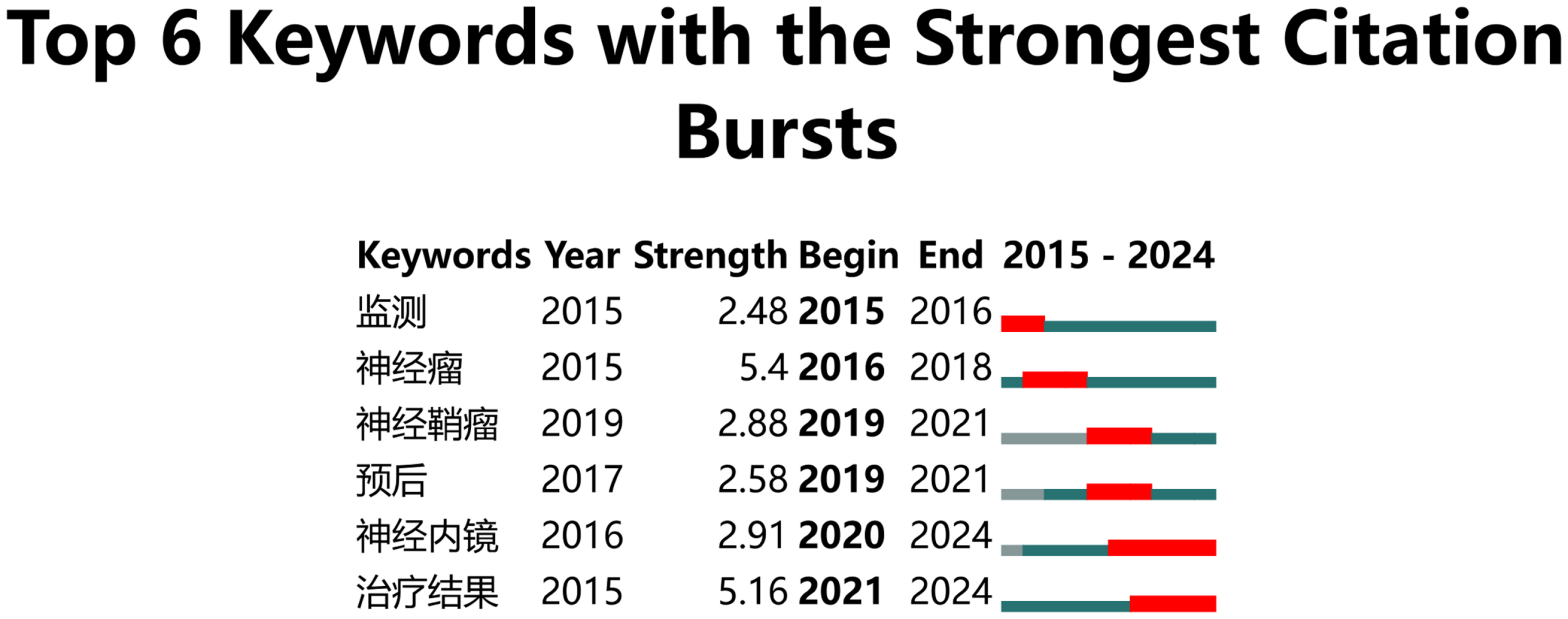
Chinese keyword mutation map.

**Figure 10:**
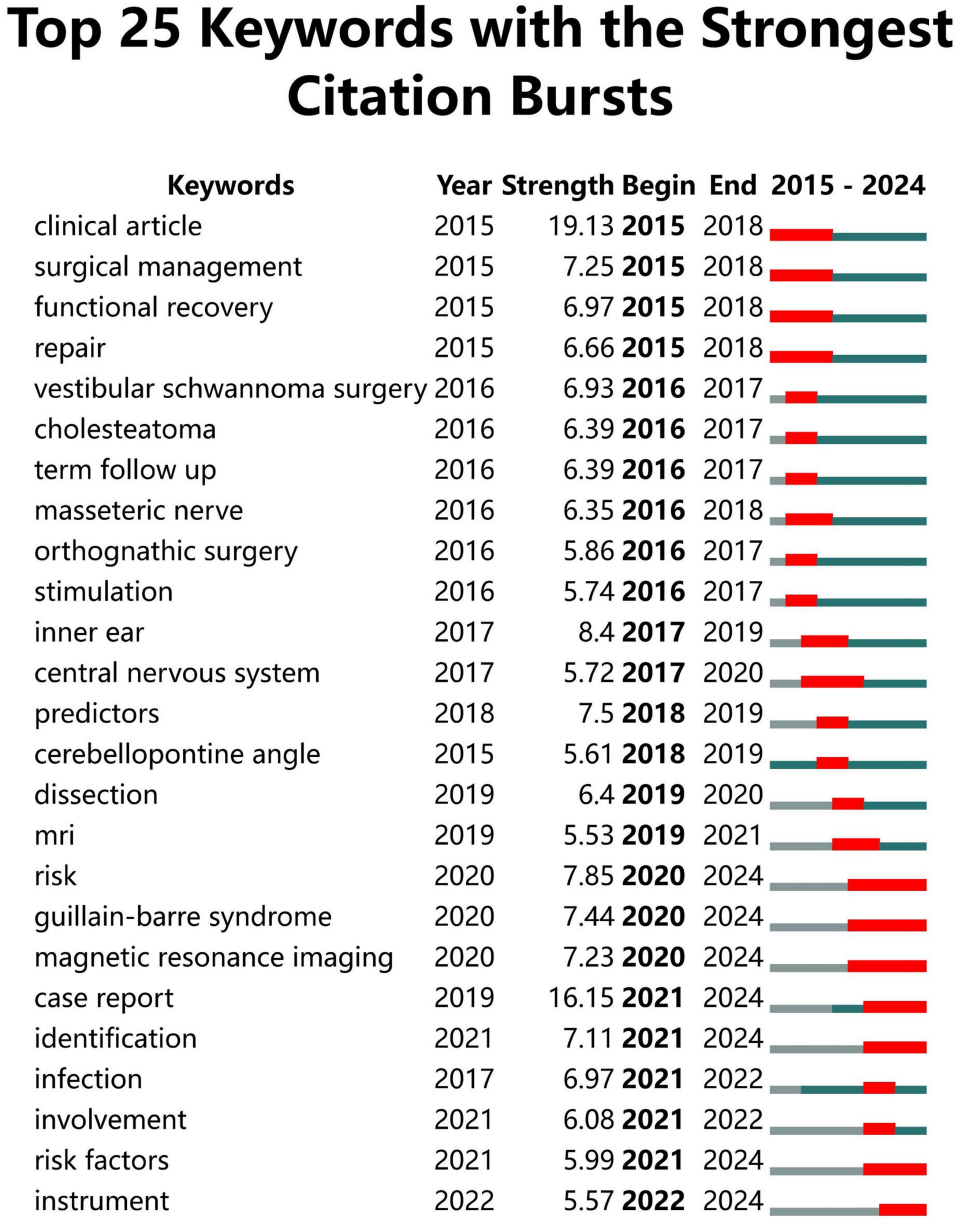
English keyword mutation map.

CiteSpace uses emerging words to reflect trends in the field (20). There were two breakout terms, “neuroendoscopy” and “treatment outcome,” that persisted to the present when the *γ* value was set to 1 in Chinese literature. A total of 39 breakout terms appeared, with 7 of the 25 most emergent mutation terms persisting to the present, namely “risk,” “Guillain-Barré syndrome,” “magnetic resonance imaging,” “case report,” “identification,” “risk factors,” and “tools.” Figures 9, 10 present the keyword mutation maps in English and Chinese, respectively.

## 4 Discussion

### 4.1 Current status of domestic and international research on facial nerve injury after CPA lesions

The results showed that domestic and international research related to nerve injury after CPA lesion has grown rapidly year by year. The longitudinal development in a specific field is reflected by the number of articles published in different years (21). In this study, the number of English studies showed an upward trend overall, increasing from 436 in 2015 to 593 in 2024. The most obvious increases were in 2019 and 2021, indicating that foreign scholars paid more attention to facial nerve injury after CPA lesions in these 2 years. This is likely due to the increase in the incidence of vestibular schwannoma (22). In addition, with the development of treatment techniques, intraoperative neuroendoscopy (23), electrophysiological monitoring (24), and magnetic imaging technology (25) have improved the identification and management of these lesions, as well as enabled more accurate assessment of facial nerve injury incidence. There has been increasing concern regarding complications, especially facial nerve injury, which is likely the reason for the rapid development and increase in relevant research. Compared with English literature, the number of articles published in Chinese showed a more stable upward trend in 2015–2017. Conversely, there has been a downward trend since 2017, indicating that Chinese research in this field is still in its initial stages.

### 4.2 Hotspots and frontiers in facial nerve injury after CPA lesions

The results and mediated centrality of high-frequency keywords analyses in domestic and international studies showed that the hotspots focused on the levels of physiological health, clinical efficacy, complications, and management. The emergent terms in national studies included “neuroendoscopy” and “therapeutic outcomes.” Domestic research has paid more attention to improving patients’ physiological health. Risk factors, case reports, early identification, and assessment tools were included in the foreign studies’ emergent words over the last 10 years, indicating that international researchers are more inclined to focus on patients’ health management, preventive measures, and early interventions with the goal of improving patients’ overall health and quality of life. In summary, research on CPA lesion with facial nerve injury is gradually transitioning from a focus on physical health to the management of both the physical and mental health in patients.

#### 4.2.1 Relevant research on assessment tools

The facial nerve is mainly responsible for the movement of facial muscles and some sensory and glandular functions, playing an important role in facial expression, taste, and glandular secretion (26). It is crucial for patients that an appropriate assessment tool be selected to accurately evaluate facial nerve function and extend of damage, which may improve treatment selection and outcomes as well as patient prognosis. The main methods used to assess facial nerve function include the House-Brackmann (H-B) Facial Function Rating Scale (27), Burres-Fisch Facial Nerve Scoring System (28), Sunnybrook Facial Nerve Rating Scale (29), Facial Nerve Function Evaluation System (30), and Quantitative Rating Scale for Facial Palsy Symptoms and Signs and so on (31). All the above scales can be used to easily and quickly assess facial nerve function and recovery in a clinical environment. The H-B Scale can be widely used in the clinic as it allows a rapid assessment and facilitates the comparison of data between different institutions. Despite all these advantages, this scale focuses only on facial muscle movement and does not adequately reflect facial nerve function. The Burres-Fisch Facial Nerve Score encompasses multiple facial movements but is susceptible to user bias. The Sunnybrook Facial Nerve Rating Scale is indicated for evaluating a wide range of facial nerve dysfunctions. The Facial Nerve Function Scoring System includes a statistical and dynamic view, and can be more objective, comprehensive, accurate, and universally applicable than the Sunnybrook System. The Quantitative Facial Palsy Symptoms and Signs Rating Scale, which covers the signs and symptoms of the facial nerve, is more complex than other rating systems and can take longer times and its results are more dependent on the assessor’s experience. With the gradual maturation of artificial intelligence (AI) technology, several AI-based facial nerve function assessment systems have been developed. The eFace, a comprehensive facial function scoring system proposed by American researchers, has been widely used (32). Existing facial nerve function assessment tools primarily focus on the motor function of the facial nerve and pay less attention to sensory and glandular secretory function. Therefore, these tools cannot comprehensively and accurately assess the degree of facial nerve damage in patients. A comprehensive assessment of facial nerve function can be developed and implemented with the help of an AI system when conditions are favorable. Meanwhile, clinical nurses need to closely monitor postoperative facial nerve function in patients with CPA tumors to identify the early occurrence of facial paralysis as a pre-sentinel symptom. This can, in turn, help them carry out functional rehabilitation training through methods such as facial muscle exercises or acupuncture.

#### 4.2.2 Management of postoperative facial paralysis, complications, and related outcomes are an important part of relevant studies

The facial nerve plays a crucial role in various physiological functions, including motor coordination, sensory perception, and parasympathetic control. Additionally, it is more susceptible to irreversible damage or even adverse outcomes, such as facial paralysis, during surgical resection of CPA tumors (26). The consequences of this injury extend beyond the physical realm and into the psychological realm, with patients facing not only a tangible loss of facial activity but also the profound challenge of impaired social expression (33). Therefore, existing studies have shifted from maximizing tumor resection and control to reducing postoperative neurological deficits by introducing intraoperative measures such as electrophysiological monitoring (24) and magnetic resonance imaging (34), especially for the facial and auditory nerves. Zhang et al. (35) used diffusion tensor imaging (DTI) preoperatively to predict the anatomical relationship between the facial nerve and vestibular schwannoma. Moreover, they protected the facial nerve intraoperatively under the guidance of neurophysiological monitoring, which reduced the risk of intraoperative facial nerve injury and improved the preservation of facial nerve function and the patients’ postoperative quality of life. However, 16 to 25% of patients still present with facial paralysis after surgery (14, 36). If patients do not undergo timely rehabilitation during the optimal recovery period following facial paralysis, or fail to regain normal facial nerve function within 12 months, the probability of long-term facial paralysis will remain high (37, 38). In severe cases, facial Synkinesis may emerge. Although there is limited research on how to help patients with facial nerve injuries rehabilitate and promote nerve function recovery. Fortunately, some studies have shown (39) that early rehabilitation can be facilitated by a multidisciplinary team using physical measures such as mirror therapy (40) or virtual reality technology (41) under the guidance of a physical therapist or rehabilitation technician. A quantity of evidences (42–44) suggests that patients with moderate or severe facial injuries exhibit higher levels of social impairment, poorer health ratings, and lower quality of life. This is supported by Keishi Fujiwara et al. (45), whose study showed that the quality of life of nearly half of the facial paralysis patients were severely compromised, particularly in terms of facial comfort and eye comfort. Thus, appropriate referral and systematic follow-up evaluations are essential components of symptom management. However, there are fewer studies on social participation, mental health, and quality of life in facial nerve injury patients in the field of nursing, which have not caused the widespread concern among researchers (8, 46, 47). This suggests that healthcare workers should consider the lower quality of life and poorer mental health of patients while assessing the degree of postoperative facial nerve injury in clinical work and scientific research.

## 5 Limitations and prospects

This study only searched for relevant literature published in the last 10 years in the CNKI and WanFang databases and Web of Science, and did not include a comprehensive search of domestic and international databases. This may have introduced bias in the reporting of results. The retrieved literature was analyzed only for authors, author institutions, and keywords. Thus, broader research results were not acquired due to the limitations of the CiteSpace software. The results of this study showed that existing studies have focused more on the therapeutic effects of different surgical or therapeutic modalities and on how to protect the facial nerve in the clinical settings. Moreover, most of the studies were predominantly focused on clinical research. The incidence of postoperative facial nerve injury in CPA lesions is high and seriously affects patients’ quality of life and mental health. There is a lack of research on facial nerve injury, particularly in the field of nursing. Moreover, existing research tools are insufficiently comprehensive for assessing facial nerve function. A more comprehensive tool can be developed in the future to identify the cluster of pre-sentinel symptoms, which can help nurses identify and intervene early, promoting the early recovery of facial nerve function. More attention should be paid to the psychological health and functional rehabilitation of patients who develop facial paralysis after CPA surgery. It is necessary to conduct interventional studies at the psychosocial level to explore the patients’ sense of shame and self-esteem, which is likely to help patients better integrate into society.

## 6 Conclusion

We identified and analyzed studies on facial nerve function related to CPA tumors over the last 10 years by searching Web of Science, as well as the CNKI and WanFang databases, using the CiteSpace 6.3 software. The results showed that existing studies are predominantly focused on clinical research, emphasizing the preservation of intraoperative facial nerve function, with less attention on the psychosocial well-being of patients and functional rehabilitation of their facial nerve in nursing care. In the future, it will be important to focus on managing of patients’ facial nerve function. Simultaneously, the efficiency of early identification and health management can be improved using new technologies such as AI and big data analysis, thereby enhancing the accuracy and effectiveness of assessment tools. Healthcare professionals also need to strengthen patient health education to improve patients participation in their own health management and enhance their understanding and ability to cope with problems.

## Data Availability

Publicly available datasets were analyzed in this study. This data can be found here: https://pan.baidu.com/s/1s4i1BAbEVc0hVpFVHkaCtw?pwd=erzs.
